# Involvement of Oxidative Stress in Occurrence of Relapses in Multiple Sclerosis: The Spectrum of Oxidatively Modified Serum Proteins Detected by Proteomics and Redox Proteomics Analysis

**DOI:** 10.1371/journal.pone.0065184

**Published:** 2013-06-07

**Authors:** Ada Fiorini, Tatiana Koudriavtseva, Elona Bucaj, Raffaella Coccia, Cesira Foppoli, Alessandra Giorgi, M. Eugenia Schininà, Fabio Di Domenico, Federico De Marco, Marzia Perluigi

**Affiliations:** 1 Department of Biochemical Sciences, Sapienza University of Rome, Rome, Italy; 2 SSD Neurology, the Regina Elena National Cancer Institute, Rome, Italy; 3 Laboratory of Virology, the Regina Elena National Cancer Institute, Rome, Italy; 4 CNR Institute of Molecular Biology and Pathology, Rome, Italy; University of Jaén, Spain

## Abstract

Multiple sclerosis (MS) is an autoimmune inflammatory demyelinating disease of the central nervous system. Several evidences suggest that MS can be considered a multi-factorial disease in which both genetics and environmental factors are involved. Among proposed candidates, growing results support the involvement of oxidative stress (OS) in MS pathology. The aim of this study was to investigate the role of OS in event of exacerbations in MS on serum of relapsing-remitting (RR-MS) patients, either in relapsing or remitting phase, with respect to serum from healthy subjects. We applied proteomics and redox proteomics approaches to identify differently expressed and oxidatively modified proteins in the low-abundant serum protein fraction. Among differently expressed proteins ceruloplasmin, antithrombin III, clusterin, apolipoprotein E, and complement C3, were up-regulated in MS patients compared with healthy controls. Further by redox proteomics, vitamin D-binding protein showed a progressive trend of oxidation from remission to relapse, respect with controls. Similarly, the increase of oxidation of apolipoprotein A-IV confirmed that levels of OS are elevated with the progression of the disease. Our findings support the involvement of OS in MS and suggest that dysfunction of target proteins occurs upon oxidative damage and correlates with the pathology.

## Introduction

Multiple sclerosis (MS) is an autoimmune inflammatory demyelinating disease of the central nervous system. In most MS patients, the disorder is characterized by a relapsing–remitting (RR) course [1]. During relapses new symptoms can appear and old ones resurface or worsen. The relapses are followed by periods of remission, during which the person fully or partially recovers from the deficits acquired during relapse. However, a neurodegenerative process leading to axonal loss and matrix destruction takes place over the course of years, and is implicated in sustained, irreversible neurological disability. Neuronal loss is more prominent when the disease takes a progressive course after years of RR episodes (secondary progressive, SPMS) or when clinical manifestations are progressive from onset (primary progressive, PPMS) [2]. The plurality of physio-pathological processes characterizing the disease, including inflammation, demyelination, and axonal damage among others, and the fact that they are not equally represented in MS population, determine the large heterogeneity in phenotypic expression of MS. The heterogeneity is present not only among the various MS forms (RRMS, SPMS, PPMS) but also within the same subtype, such as between the relapse and remission in RRMS. So far, what determines the exacerbation of the disease, as well as biomarkers able to give information about its progression, is yet unknown.

Since MS is considered not only an inflammatory disease but also a neurodegenerative disorder [3], [4], many evidences support the crucial role of oxidative stress (OS) in the pathogenesis of MS [5], [6]. Several studies highlight the presence of oxidative damage both in blood and in the nervous system of patients with MS [7], [8]. Increased protein carbonyls were found in post-mortem brains of MS patients [9], in addition to elevated contents of OS markers in CSF and plasma from MS patients [6], [9], [10]. Further, Tasset et al. [11] showed a significant peripheral OS in RRMS patients.

The recent understanding of the patho-physiology of MS has led to the development of many drugs that counter exacerbations and the formation of new lesions in patients with relapsing remitting MS. Presently there are no therapies able to reduce neurodegenerative damages [12], therefore it is urgently needed to find new cures to contrast the progression of disability in MS.

Proteomic analysis is a powerful tool to identify putative biomarkers, to elucidate the molecular mechanisms underlying the disease, to allow monitoring of disease progression, and to find putative therapeutic targets for the treatment of the disease [13]–[15].

So far, most of the proteomic studies of patients affected by MS have been performed on cerebrospinal fluid (CSF) considering exclusively a single form of MS patients or a combined group of all subtypes of MS [16]–[18]. Recently, Stoop et al. [19] performed a proteomics study comparing CSF between RR and PP MS. Teunissen et al. [18] found novel potential biomarkers of the disease progression using proteomics technologies in CSF and serum of patients affected by all of three MS forms (RR, PP, SP). In addition, in the same study biomarkers discriminating patients with MS from others affected by different nervous disorders are shown.

In the present study, we aimed to describe the proteomic profile of the low-abundant serum protein fraction of MS patients, each one in remitting and in relapsing phase to reduce the inter-individual variability. MS patients were also compared with the sex and age-matched control group. Serum is a promising resource for biomarkers discovery, despite its complex nature and the presence of high abundant proteins masking subtle changes in the less abundant ones, can make disease biomarkers detection a very challenging task. Therefore, depletion of the most abundant proteins is an important step towards improving the analysis of serum.

In particular, in order to shed light on the role of OS in MS, we focused our attention to identify differently expressed and oxidatively modified proteins, by applying proteomics and redox proteomics approaches. Oxidative post-translational modification of proteins may produce structural changes in proteins that could be reflected on their functions, and therefore identification of these proteins could be useful in understanding which pathways are impaired in MS. The implications and the conceivable impact of the identified proteins on the molecular mechanisms and clinical outcomes of MS are discussed.

## Materials and Methods

Study population was composed by patients with diagnosis of MS according to the revised McDonald criteria [20] enrolled at Multiple Sclerosis Centre, Regina Elena National Cancer Institute of Rome (Italy), and the sex- and age- matched healthy controls. The study was approved by Ethic Committee of Regina Elena National Cancer Institute and an informed written consent was obtained by all the participants.

### Sample Collection

Blood samples from the same 18 RRMS patients in their relapsing (Group I) or remitting phase (Group II) and 7 healthy controls (Group III) were evaluated. A definition of MS relapse adopted was the modified Schumacher et al criteria [21]. with a minimum of 48 hours of symptom duration as well as changes in functional measures, as assessed using the Expanded Disability Status Scale (EDSS).

At the day of sampling all patients were assessed for physical and cognitive performance with followed examinations: EDSS; Multiple sclerosis severity Score (MSSS); Fatigue Severity Scale (MFIS); Beck Depression Inventory (BDI); Paced Auditory Serial Addition Test (PASAT). All patients were treated with immunomodulatory therapy in the previous 3 months. Subjects’ characteristics are listed in Table 1.

**Table 1:** Demographic and clinical characteristic of multiple sclerosis patients.

	Age	M/F	EDSS	Dis Dur	MSSS	MFIS	BDI	PASAT
***Group I (Relapsing)***	43.2±10	5/13	3.3±1.1	7.4±6	5.3±1.8	35.6±16.6	13.5±9.9	42.9±11.7
***Group II (Remitting)***	43±10.1	5/13	2.3±1.5	7.4±6.4	3.0±2.0	33.2±19.4	10.6±8.6	44.3±24.0
***Group III (Control)***	46.2±11.7	3/4	6.7±0.8					

EDSS = Expanded Disability Status Scale; Dis Dur = Disease duration; MSSS = Multiple sclerosis severity Score; MFIS = FatFatigue Severity Scale; BDI = Beck Depression Inventory; PASAT = Paced Auditory Serial Addition Test.

Re Reported values are ± SD.

### Sample Preparation

Serum samples from MS patients were grouped into pools (six pools, each with three samples) to reduce the individual and biological variability that has been reported in plasma proteins [22]. Though this step involves the loss of each sample individual data, this pooling strategy has demonstrated its usefulness for biomarker discovery in shotgun proteomics approaches [23]. Serum from healthy controls were processed individually (n = 7).

In order to improve detection of low-abundant proteins, serum samples were purified with MARS-14 Column (Agilent Tech.), which removes 95 percent to 99 percent of the 14 most abundant proteins. These high-abundance proteins account for 94 percent of the total protein mass, and depletion of those facilitates the discovery and the identification of low-abundance proteins. Proteins from either the six pools, for each MS group, and the seven CTR samples were purified on a MARS-14 column and eluted according to the manufacturer’s instructions. Protein determination was performed on the eluate fraction by the Bradford protein assay, using bovine serum albumin as a standard.

### Measurement of Protein Carbonyls (PC)

Protein carbonyl levels were detected as adducts of 2,4-dinitrophenylhydrazine (DNPH). Five microliters of the samples (corresponding roughly to 2 µg of proteins) were treated with an equal volume of 12% SDS. Samples were then derivatized with 10 µL of 20 nM 2,4-DNPH in 2 N HCl for 20 min. After, samples were neutralized with 7.5 µL of 2 M Tris/30% glycerol buffer, pH = 8.0. Levels of protein carbonyls were measured by using the slot-blot technique with 250 ng of protein loaded per slot. The 2,4-dinitrophenylhydrazone (DNP) adducts were detected on the nitrocellulose paper using a primary DNP specific rabbit antibody (Millipore) (1∶100) followed by a secondary goat alkaline phosphatase-conjugated anti-rabbit IgG (Sigma Aldrich). The reaction product was revealed by 5-Bromo-4-chloro-3-indolyl phosphate dipotassium combined with Nitrotetrazolium Blue chloride (BCIP/NBT) in ALP buffer [0.1 M Tris, 0.1 M NaCl, 5 mM MgCl_2_ 6 H_2_O (pH 9.5)]. After developing, blots were allowed to dry overnight, scanned on a GS800 densitometer (Biorad), and quantified by QuantityOne image software.

### Two-Dimensional(2D) Gel Electrophoresis

Eluted proteins (100 µg) for each sample were diluted to a total volume of 200 µl with rehydration buffer (8 M urea, 20 mM dithiothreitol (DTT), 2.0% (w/v) Chaps, 0.2% Bio-Lyte, 2 M thiourea and bromophenolblue), and placed in agitation for 3 hours. For the first-dimension electrophoresis, 200 µL of sample solution were applied to a ReadyStrip ™IPG strip pH 4–7 (Bio-Rad). The strips were soaked in the sample solution for 1 h to allow uptake of the proteins. The strips were then actively rehydrated in a Protean IEF Cell Apparatus (Bio-Rad) for 16 h at 50 V. The isoelectric focusing was performed at 300 V for 2 h linearly; 500 V for 2 h linearly; 1000 V for 2 h linearly, 8000 V for 8 h linearly and 8000 V for 10 h rapidly. All the processes above were carried out at room temperature. The focused IEF strips were stored at –80°C until second dimension electrophoresis was performed.

For second dimension electrophoresis, the strips were equilibrated for 10 min in 50 mM Tris-HCl (pH 6.8) containing 6 M urea, 1% (w/v) sodium dodecyl sulfate (SDS), 30% (v/v) glycerol, and 0.5% dithiothreitol (w/v), and then re-equilibrated for 15 min in the same buffer containing 4.5% iodacetamide in place of dithiothreitol. 12% Precast criterion gels (Bio-Rad) were used to perform second dimension electrophoresis. After electrophoresis, the gels were incubated in fixing solution (7% acetic acid, 10% methanol) for 45 min, and then stained for 1 h in Bio-Safe Coomassie gel stain (Bio-Rad) and destained overnight in deionized water. The Coomassie gels were scanned using a GS 800 densitometer (Bio-Rad).

### Immunochemical Detection

For immunoblotting analysis, electrophoresis was carried out in the same way as previously described, and the gels were transferred to nitrocellulose membranes. Six blots, corresponding to each single pool, for each MS group and seven blots from CTR samples were performed. To identify carbonylated proteins, blots were derivatized with 2,4-dinitrophenilhydrazine (DNPH). Briefly, membranes were equilibrated in 20% methanol (5 min), then incubated in 2 N HCl (5 min), and finally derivatized in 0.5 mM DNPH solution (5 min). After derivatization, three washes using 2 N HCl solution and five washes using methanol 50% were performed (5 min each). The membranes were then blocked with 3% albumin in T-TBS, incubated with the primary Rabbit x DNP antibody (1∶100; Millipore) followed by the secondary antibody alkaline phosphatase-conjugated anti-rabbit IgG (1∶5000; Sigma) and revealed by 5-Bromo-4-chloro-3-indolyl phosphate dipotassium combined with Nitrotetrazolium Blue chloride (BCIP/NBT) in ALP buffer [0.1 M Tris, 0.1 M NaCl, 5 mM MgCl_2_ 6 H_2_O (pH 9.5)].

### Image Analysis

2D gels (a total of 12 from MS patients and 7 from CTR) and 2D blots were analyzed by PDQuest 2D Analysis (7.2.0 version; Bio-Rad). PDQuest spot-detection software allows the comparison of 2D gels as well as 2D blots from different groups. Powerful auto-matching algorithms quickly and accurately match gels or blots and sophisticated statistical analysis tools identify experimentally significant spots. The intensity value for each spot from an individual gel is normalized using the average mode of background subtraction. This intensity is afterward compared between groups using statistical analysis. Statistical significance was assessed using a two-tailed Student t-test. P values <0.05 were considered significant for comparison between control and MS patients data. In order to confirm data from gel comparison, each analysis was repeated three times by setting a different master gel every time according to the software instructions.

PDQuest software allows also normalization of a carbonylated spot intensity on the blot versus the expression level of the same spot on the gel. One-dimensional blots were analyzed with QuantityOne software (4.6.9version;Bio-Rad).

### Trypsin Digestion and Proteins Identification by Mass Spectrometry

Selected spots were manually excised from gel and submitted to trypsin proteolysis [24]. MALDI-ToF MS analyses were performed with an AutoFlex II instrument (Bruker Daltonics, Bremen, Germany), equipped with a 337 nm nitrogen laser and operating in reflector positive mode. Two tryptic autolytic peptides were used for the internal calibration (m/z 842.5100 and 2807.3145). Data were analyzed by flex Analysis program (Bruker Daltonics, Bremen, Germany). Identification by peptide mass fingerprint (PMF), with the mono-isotopic mass list, was performed using Bio Tools program (Bruker Daltonics, Bremen, Germany), by the Mascot search engine, against human SwissProt database [(SwissProt 2012_10 (20234 sequences)]. Up to two missed cleavage, 50 ppm measurement tolerance, oxidation at methionine (variable modification) and carbamidomethylation at cysteine (fixed modification) were considered. Identifications were validated when the probability-based Mowse protein score was significant according to Mascot [25].

### Statistical Analysis

For comparison of values between relapsing and remitting patients and controls, data from each group were checked for normality using the Shapiro–Wilk statistical test, and if this assumption was nonviable log transformation was applied. All statistical analyses were performed using a nonparametric one-way ANOVA with post hoc *t*-test. PDQuest software was used to select a master gel representing each group. For analysis of differentially expressed proteins among matched gels, spots whose intensities were either increased or decreased 1.5-fold or greater were marked and also confirmed by manual inspection of all relevant 2D gels to ensure consistency. Quantitative analysis was performed using the Student’s *t*-test among three groups of gels. Significance was accepted if *P* value <0.05.

## Results

### Protein Carbonyl Level

**Figure 1:**
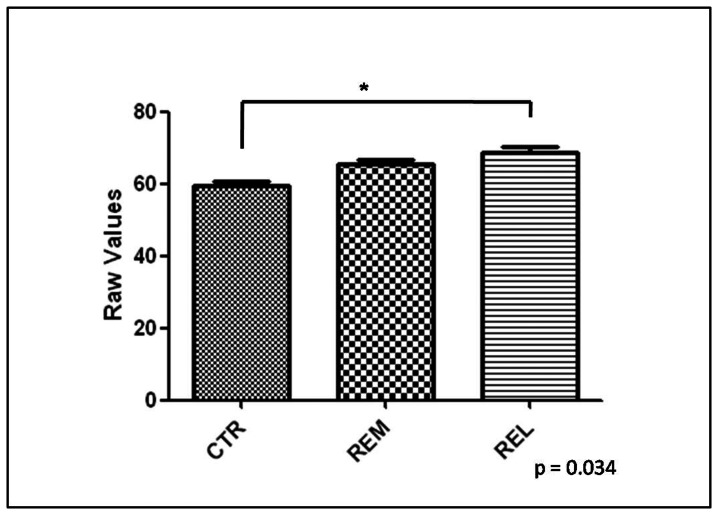
Levels of protein carbonyls. Protein carbonyls (PC) levels in the serum of control (CTR) and multiple sclerosis patients: remitting (REM) and relapsing (REL). Serum samples were assayed for PC by slot blot analysis. Error bars indicate SD for 6 samples per group. Densitometric values shown are given as raw values.

Analysis of protein carbonyl levels revealed a small, but significant increase of this OS marker in serum of MS patients in relapsing phase respect to controls (p = 0.034). There were no significant differences in protein carbonyl levels between MS patients in remitting phase respect to MS patients in relapsing phase and respect to controls. These data are shown in Fig. 1.

The same trend was also observed by analyzing the depleted fraction (14 most abundant proteins) of serum samples (Figure S1).

### Proteomics and Redox Proteomics

**Figure 2:**
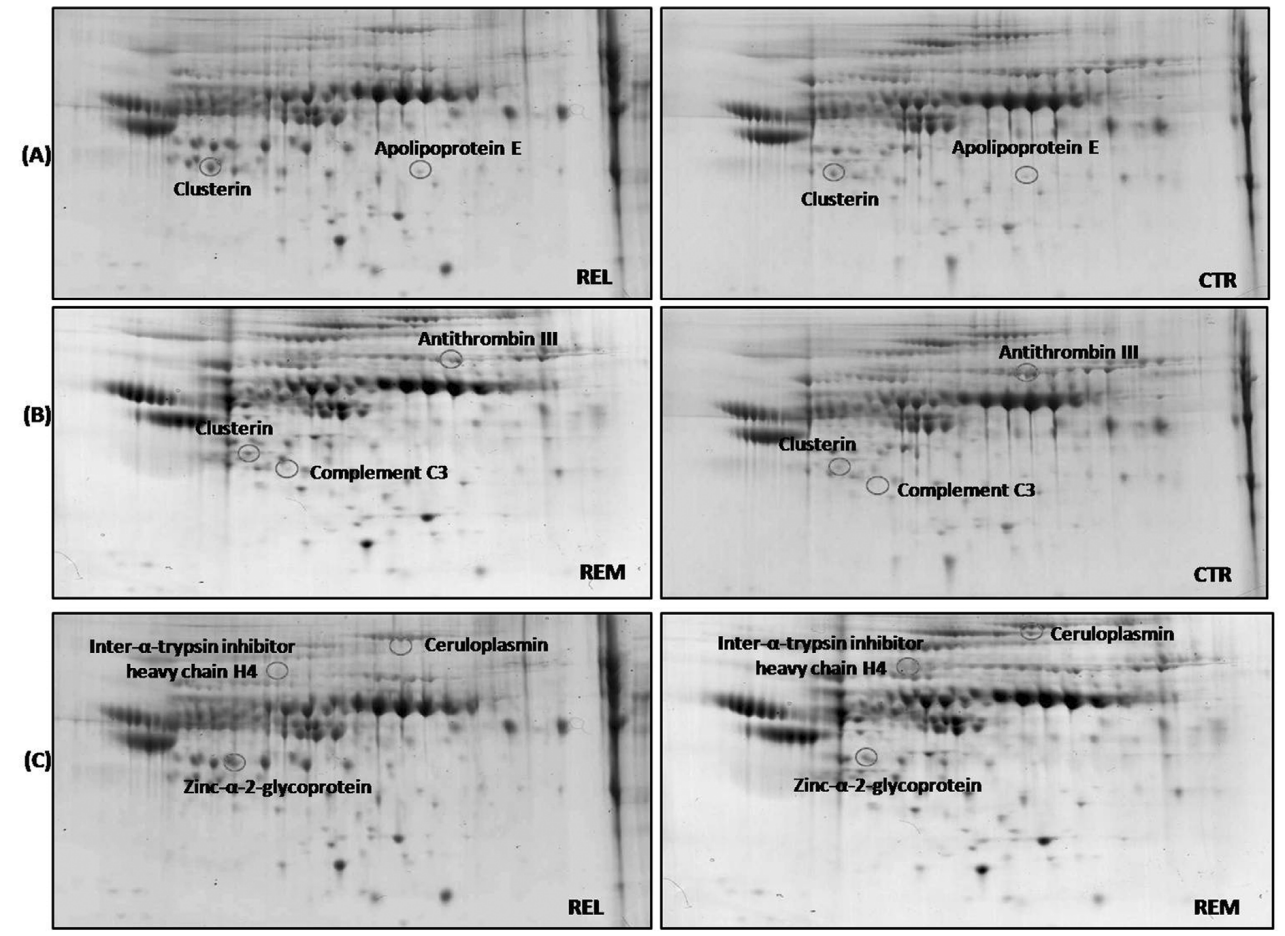
2D protein expression maps. Proteomic profile of representative 2D-gels with proteins differently expressed in the three groups of matching: REL vs CTR (A), REM vs CTR (B) and REL vs REM (C). The identified proteins by mass spectrometry are listed in Table 2.

Proteomics analysis using 2-DE and Coomassie staining were performed in serum samples of patients with MS in relapsing (REL) or in remitting phase (REM) and in control group (CTR) to determine differently expressed proteins. In Fig. 2, 2-D gel images related to the matching between REL vs CTR, REM vs CTR, and REL vs REM are shown. Additional gels from the three groups of analysis are shown in Figure S2. Eight proteins showed expression differences. These protein spots were excised from the gels, and following trypsin digestion the peptides were analyzed by MS/MS. Molecular weight (MW) and isoelectric point of all identified proteins were consistent with those shown by protein positions on the gel. These are listed in Table 2 with the number of peptide sequences, the coverage, MW, pI, fold-change levels, and p-value.

**Table 2:** Summary of the proteins identified as differently expressed using the proteomics approach.

Protein Identified	SwissProtCode	% sequence coverage	Theoretical mw/pI(kDa)	Groups	P value^a^	Fold^b^
**Ceruloplasmin**	P00450	10	122.98/5.44	Rel-Rem	0.025	6.0↑ Rem
**Inter-α-trypsin inhibitor** **heavy chain H4**	Q14624	15	103.521/6.51	Rel-Rem	0.037	3.9↑ Rel
**Zinc-α-2-glycoprotein**	P25311	32	34.465/5.71	Rel-Rem	0.049	2.8↑ Rel
**Antithrombin III**	Q9UC78	40	53.025/6.32	Rem-Ctr	0.044	3.1↑ Ctr
**Clusterin**	P10909	24	53.031/5.89	Rem-Ctr	0.034	4.0↑ Rem
**Clusterin**	P10909	14	53.031/5.89	Rel-Ctr	0.038	3.6↑ Rel
**Apolipoprotein E**	P02649	45	36.246/5.65	Rel-Ctr	0.017	1.8↑ Rel
**Complement C3**	P01024	7	188.569/6.02	Rem-Ctr	0.034	1.5↑ Rem

a The p-value associated with fold-change calculated using a Student’s t-test.

b The fold-change in spot density from three groups of matching: Rel vs Ctr; Rem vs Ctr; Rel vs Rem. The arrow indicates the direction of change.

From the matching between REL vs CTR, two proteins were found to be differently expressed: clusterin and apolipoprotein E, both of which showed to be over-expressed in REL compared to CTR (3.6-fold, and 1.8-fold, respectively).

In the analysis of REM vs CTR we identified three proteins: clusterin and complement C3 were up-regulated in REM compared to CTR (4.0-fold, and 1.5-fold, respectively), while antithrombin III was down-regulated in REM with respect to CTR (3.1-fold).

The match between REL vs REM showed three differently expressed proteins: the inter-α-trypsin inhibitor heavy chain H4 and the zinc-α-2-glycoprotein were, up-regulated (3.9-fold, and 2.8-fold, respectively), and ceruloplasmin was down-regulated (6.0-fold).

**Figure 3:**
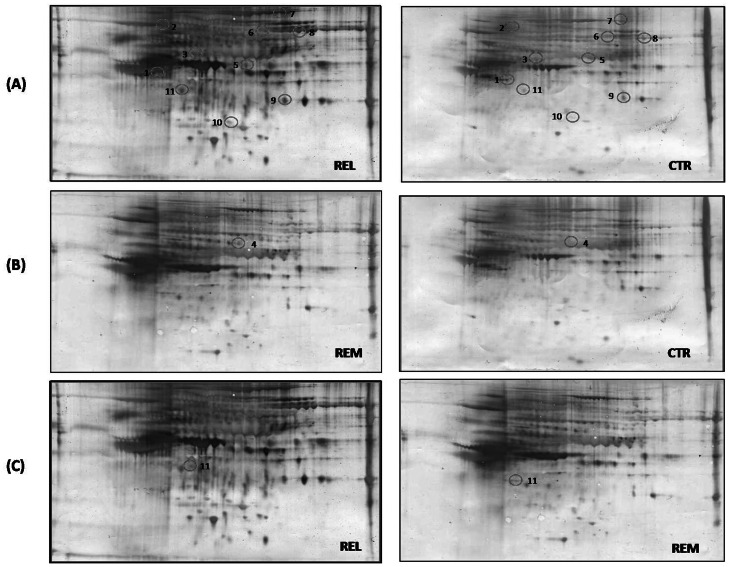
Oxidized protein detection by redox proteomics. Proteomic profile of representative 2D-blots with proteins differently oxidized in the three groups of matching: REL vs CTR (A), REM vs CTR (B) and REL vs REM (C).The identified proteins are listed in Table 3.

Specific carbonylated proteins were detected immunochemically using 2D-gels and 2D-Western blots. 2D-gels and 2D-blots probed with DNP- antibody were matched using the PD-Quest software and the specific protein carbonyl levels were obtained by dividing the protein carbonyl level of a single spot on the blot by the protein level of its corresponding protein spot on the gel. Fig. 3 shows three representative 2D gels and the corresponding 2D blots of REL vs CTR, REM vs CTR, and REL vs REM.

Relative change in carbonyl immune-reactivity, after normalization of the immunostaining intensities to the protein content, was significant for eleven spots. After spot excision and in-gel trypsin digestion, proteins were identified by MS/MS analysis.

In Table 3, the carbonylated proteins identified by MS/MS and interrogation of databases are listed. Interestingly, most of these proteins were more oxidized in relapsing patients compared with control patients. This result confirms our data about the measure of total levels of carbonylated proteins.

**Table 3:** Summary of the proteins identified as differently oxidized using the redox proteomics approach.

Protein Identified	Spot	SwissProt code	% sequence coverage	Theoreticalmw/pI (kDa)	Groups	P value^a^	Fold^b^
**Nebulin-related-anchoring protein**	1	Q80XB4	22	194.0/9.4	Rel-Ctr	0.003	115↑ Rel
**Inter-α-trypsin inhibitor** **heavy chain H4**	2	Q14624	29	103.52/6.51	Rel-Ctr	0.002	103↑ Rel
**α-1B-glycoprotein**	3	P04217	29	54.79/5.56	Rel-Ctr	0.0004	326↑ Rel
**Vitamin D-binding protein**	4	P02774	33	54.52/5.4	Rem-Ctr	0.025	65.2↑Rem
**Vitamin D-binding protein**	9	P02774	9	54.52/5.4	Rel-Ctr	0.028	213 ↑Rel
**Hemopexin**	5	P02790	23	52.38/6.55	Rel-Ctr	0.049	118↑ Rel
**Hemopexin**	7	P02790	25	52.38/6.55	Rel-Ctr	0.0004	307↑ Rel
**Antithrombin-III**	6	Q9UC78	22	53.025/6.32	Rel-Ctr	0.019	305↑ Rel
**Gelsolin**	8	P06396	21	86.04/5.9	Rel-Ctr	0.023	215↑ Rel
**Serum amyloid P-component**	10	P02743	27	25.48/6.1	Rel-Ctr	0.012	841↑ Rel
**Apolipoprotein A-IV**	11	P06727	60	45.37/5.28	Rel-RemRel-Ctr	0.0120.012	23↑ Rel346↑ Rel

a The p-value associated with fold-change calculated using a Student’s t-test.

b The fold-change in spot density from three groups of matching: Rel vs Ctr; Rem vs Ctr; Rel vs Rem. The arrow indicates the direction of change.

From the matching between REL vs CTR the identified proteins were: nebulin-related-anchoring protein (115-fold increase), inter-α-trypsin inhibitor heavy chain H4 (103-fold increase), α-1B-glycoprotein (326-fold increase), hemopexin (118-fold increase and 307-fold increase), antithrombin-III (305-fold increase), gelsolin (215-fold increase), vitamin D-binding protein (DBP) (213-fold increase), serum amyloid P-component (841-fold increase), and apolipoprotein A-IV (346-fold increase).

In the analysis between REM vs CTR one protein was found more oxidized in REM compared to CTR (65.2-fold): DBP.

The match between REL vs REM showed only one protein more oxidized in REL with respect to REM: apolipoprotein A-IV (23-fold).

**Figure 4:**
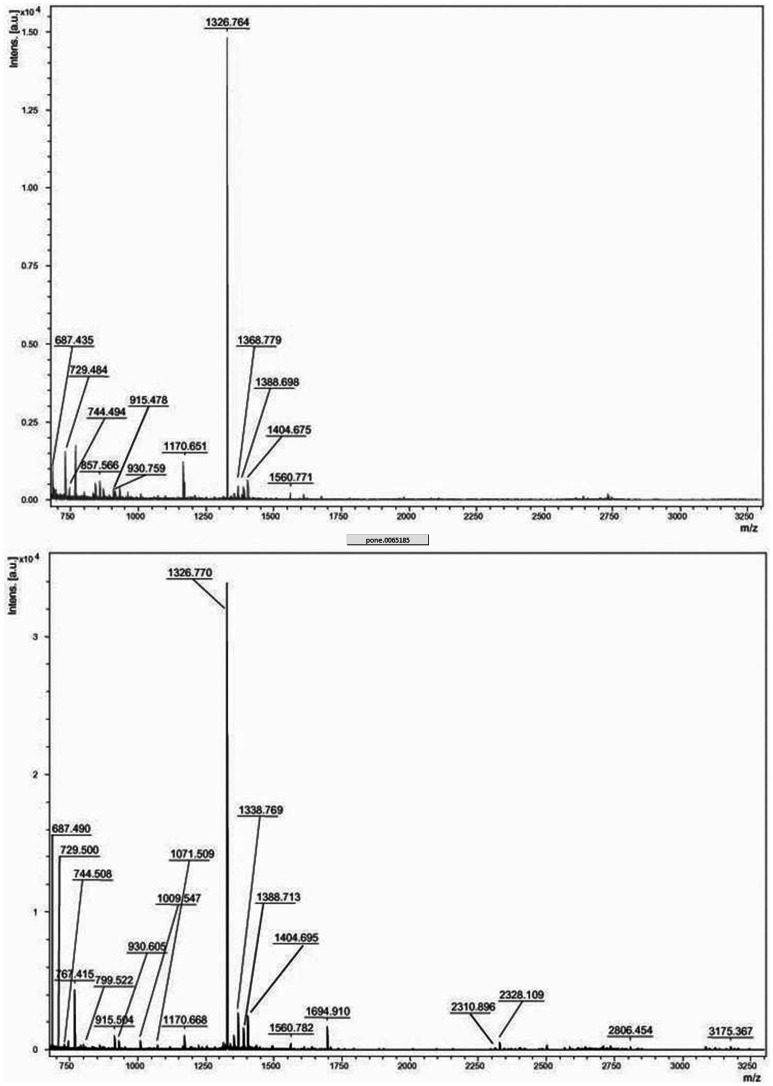
Spectra obtained by MALDI-ToF MS analysis related to spots of vitamin D-binding protein (DBP). DBP C-terminal region spectrum (upper panel) and major coverage DBP spectrum (lower panel).

As shown in Fig. 3, two spots with different MW and pI, were identified as DBP. A rational explanation to our findings is that in relapse phase the higher OS levels result in increased oxidation of the protein which also causes its subsequent fragmentation [26]. Indeed, in the matching between REL vs CTR solely C-terminal portion of DBP was revealed by mass spectrometry. To corroborate our notion, spectra obtained by MALDI-ToF MS analysis related to spots of DBP are shown in Fig. 4. This result indicates that the two spots correspond to the same protein, DPB, which in the case of REL group was identified as a fragment, most likely due to its oxidative modification. The same consideration can be also applied to hemopexin which has been identified by two different spots as well.

**Figure 5:**
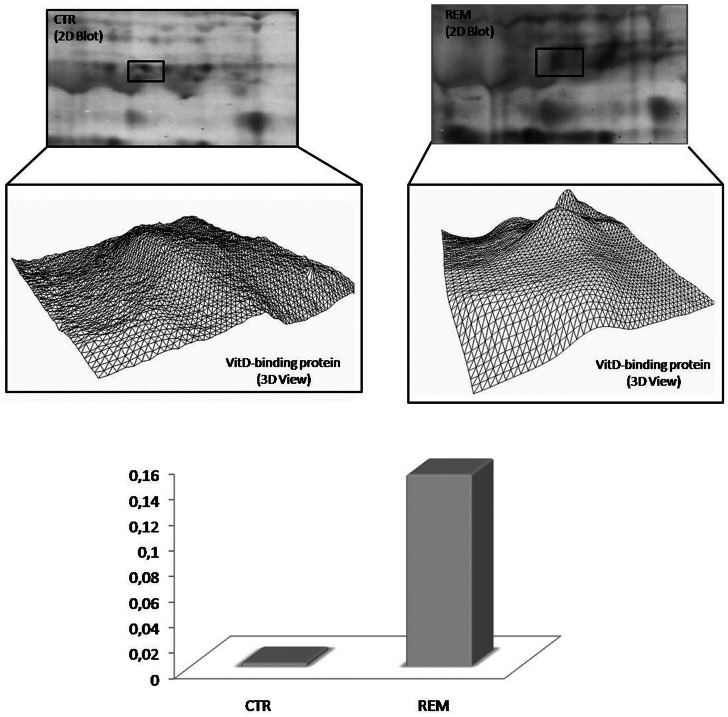
Vitamin D-binding protein oxidation fold in REM vs CTR matching. In the upper part of the figure, an enlarged area of 2D blots from CTR and REM groups is shown corresponding to the vitamin D-binding protein (DBP). DBP identified as differently oxidized in the matching between REM vs CTR is labeled. 3D density graphs are elaborated by PD-Quest from DBP spot on 2D blot. In the lower part of the figure the histogram reports oxidation fold for DBP.

**Figure 6:**
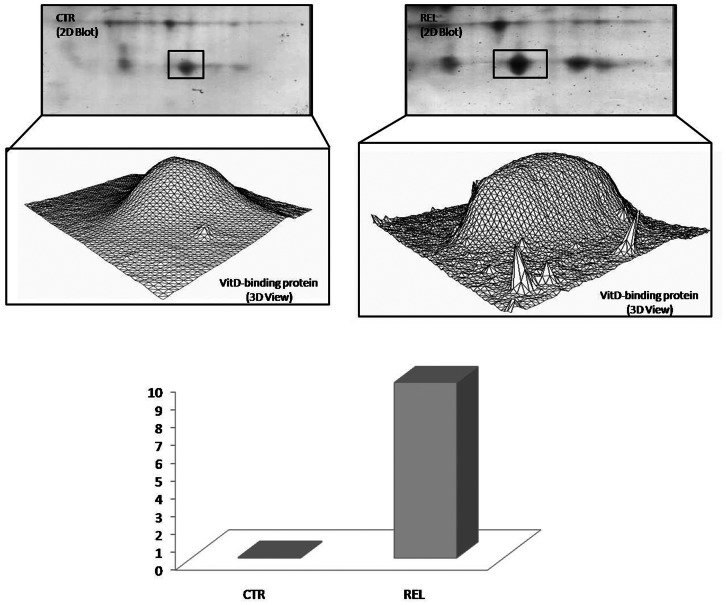
Vitamin D-binding protein oxidation fold in REL vs CTR matching. In the upper part of the figure, an enlarged area of 2D blots from CTR and REL groups is shown corresponding to the DBP. DBP identified as differently oxidized in the matching between REL vs CTR is labeled. 3D density graphs are elaborated by PD-Quest from DBP spot on 2D blot. In the lower part of the figure the histogram reports oxidation fold for DBP. (Data obtained from Proteomics and redox proteomics approach).

DBP seems to be the only protein differently carbonylated in both relapsing patients compared to controls (p = 0.028), and in remitting patients compared to controls (p = 0.025). In either matching, DBP is more oxidized in pathological samples, as shown in Fig. 5 and in Fig. 6.

**Figure 7:**
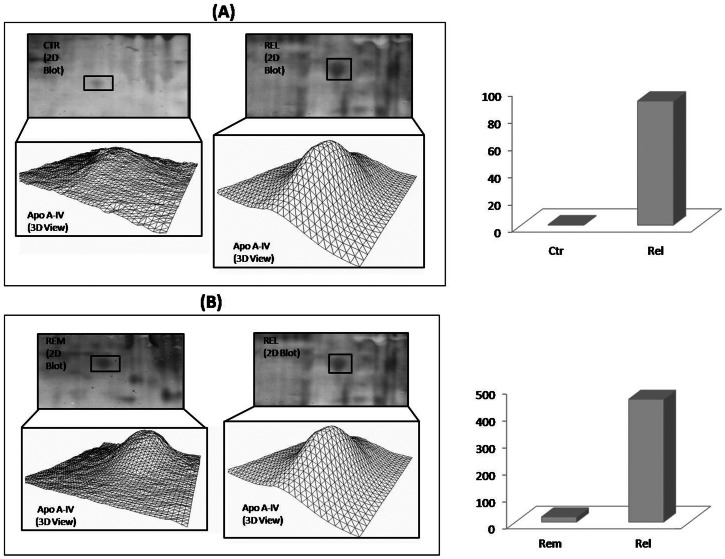
Apolipoprotein A-IV oxidation fold. On the left side of the figure, an enlarged area of 2D blots from CTR and REL groups (A), and from REM and REL (B),are shown corresponding to the Apolipoprotein A-IV (Apo A-IV) spot. Apo A-IV spot identified as differently oxidized in the matching between REL vs CTR, and REL vs REM is labeled. 3D density graphs are elaborated by PD-Quest from Apo A-IV spot on 2D blot. On the right side of the figure the histogram reports oxidation fold for Apo A-IV.

Another protein, namely apolipoprotein A-IV, was found more oxidized in relapsing group compared to both control and remitting group (Fig. 7).

The proteins identified as differently expressed or oxidized are listed in Table 4 correlated with their functions.

**Table 4:** Functions of Identified Proteins Differently Expressed and Oxidized.

Functions	Proteins involved
**Inflammatory response**	Hemopexin; Alpha-1-B glycoprotein; Inter-α-trypsin inhibitor heavy chain H4;Complement C3
**Thrombin inhibition**	Antithrombin III
**Innate response**	Serum amyloid P-component
**Actin-binding**	Nebulin-related anchoring protein; Gelsolin
**Lipid metabolism**	Apoliprotein A-IV; Apolipoprotein E; Zinc-α-2-glycoprotein
**Vitamin D carrier; Actin binding; Immune response**	Vitamin D-binding protein
**Chaperone-activity**	Clusterin
**Fe homeostasis**	Ceruloplasmin; Hemopexin

## Discussion

The etiology of MS is not yet completely known. Growing results support a pivotal role of OS in MS pathogenesis and progression [5], [27]. Increased levels of OS markers and decreased levels of antioxidant molecules have been described in patients with MS [6]. This imbalance has been implicated in demyelination and axonal damage and a positive correlation between OS markers and expanded disability status scale was recently observed [28]. Pathological and clinical data collected in the past decade reveale that MS pathological mechanisms may vary according to the stage of the disease [29]. It is likely that there is on-going OS during different phases of MS. Recently the concept of MS as a biphasic disease has been divided into an inflammatory RR phase and a degenerative SP phase [30]. However, it is not clear how OS contributes to the occurrence of relapse in RRMS patients or eventually to further degeneration in SP. By evaluating total protein carbonyl levels in serum, we confirmed that oxidative damage is increased in relapse phase of MS patients. Indeed, we found higher protein carbonyl levels in relapsing phase compared with both remitting and healthy groups. In order to better understand this trend, we performed both proteomics and redox proteomics approaches to identify changes in serum proteome profiles in subjects of these three groups. Serum is a rich source of disease related proteins. Detecting them is extremely challenging due to the body fluid’s characteristics. One particular feature is the risk that abundant proteins may interfere with identification and quantification of the less abundant ones, which are usually recognized as putative disease biomarkers. In order to improve the detection of low abundant proteins, we depleted the most-abundant protein fraction in our serum samples.

By comparing serum proteome profile of three groups of analysis, CTR, REM and REL, we identified differently expressed proteins, obtaining results in part consistent with data already showed in literature [31], thus validating our experimental approach. Interestingly, many of these proteins were up-regulated in MS patients compared to healthy controls, and in particular clusterin showed an intriguing trend, since its expression was increased already in remitting phase and kept up-regulated in relapsing phase. Clusterin is demonstrated to be implicated in several disorders characterized by increased OS and high cell death [32]. One of the most investigate areas among its functions, is chaperone activity, thanks to which clusterin binds to partly unfolded proteins preventing their precipitation and protecting cells from the cytotoxic consequences [33]. For its protective capacity, clusterin expression has been shown to be up-regulated in cells under OS conditions [32]. Therefore, the increased expression levels of clusterin in REM and REL compared to CTR indicate that its up-regulation is an indirect “measure” of OS, which is found to occur already in remission and further increase in relapse. Thus, it could be used to assess the levels of OS in different phases of MS.

To more thoroughly investigate changes in the serum proteome, we also performed a redox proteomics analysis to reveal OS specific proteins potentially dysfunctional and linked to the pathological course of the disease.

Among our results, particularly intriguing appear the modifications shown by vitamin D-binding protein (DBP). DBP was found to be more oxidized in both the remitting and relapsing phases compared to controls, further showing a progressive rate of oxidation from control, to remission and to relapse., thus showing a full concordance with the course of the disease. DBP is the major plasma carrier protein of vitamin D metabolites [30]. It is synthesized by the liver in an estrogen dependent manner and is removed by several tissues such as kidney, skeletal muscle, intestine, bone, lung and the liver itself. The balance between production and clearance constantly maintains DBP plasmatic levels in molar excess respect to its major ligand, the 25-OH-vitamin D. A close connection between DBP level and the clinical course of MS has been recently suggested by a number of proteomic studies [29]. In their complex these studies indicate reduced DBP levels in CSF of patients with clinically isolated syndrome and RRMS relapses, while increased levels are found in patients with the progressive form [34]. The reported evidence of sharply relevant changes in DBP oxidation occurring during relapses introduce a further level of complexity in DBP activity during MS and consequently in the modulation of the whole vitamin D axis. It is to be stressed however that it is still unknown whether the role of DBP is dependent on its vitamin D binding activity, s on the other hand, it is well-established that DBP has a distinct immune-modulatory effect independent by vitamin D binding [35].

In light of the importance of immune response on the development of MS, oxidation of DBP seems to highlight the dysfunction of this protein as a selective alteration of this pathology. Moreover, the increased oxidation rate of DBP already in the remitting phase, supports the notion that some molecular pathways are not completely suppressed during remission. They may represent potential targets to reduce the severity of relapse and slowdown reoccurrence.

MS is mainly considered an inflammatory disease of the central nervous system, but several evidence further support an involvement of the coagulation system in its pathogenesis [36]. A link between the inflammation and coagulation system is well established [37], [38]. High levels of thrombin and its plasma inhibitor, AT-III [39], were found in experimental autoimmune encephalomyelitis (EAE) brains during the peak of the disease. This is due to an increase of blood-brain barrier (BBB) permeability occurring in the acute phase of EAE allowing coagulation factors and their inhibitors to enter in the CNS [40]. Moreover, it has been shown that thrombin exacerbates EAE, thus inhibiting thrombin may have potential benefit for MS treatment [41]. Based on these notions, thrombin is considered a new therapeutic target for the cure of MS, and thrombin inhibitors, such as AT-III, a possible candidate drug [41]. In our study, AT-III has been found either down-regulated in the remitting phase compared to controls or oxidatively modified in the relapse phase compared to healthy patients, reinforcing the importance of the coagulation system in MS course according to current literature. Our results suggest that the balance restoration of the coagulation system may reduce the inflammatory damage typical of MS, and also may prevent the progression of the disease.

In addition, a number of acute phase proteins including: hemopexin, α-1-B-glycoprotein, inter-α-trypsin inhibitor heavy chain H4, and complement C3 have been found to be differently expressed and more oxidized in pathological versus controls samples. These same proteins were already detected differently expressed in inflammatory disease such as mastitis [42] thus further underscoring the link between MS pathogenesis and the disruption of the inflammatory response regulation.

Recently, a new aspect of MS is emerging which supports the involvement of altered phospholipid metabolism in the etiology of the disease [43]. Detection of biomarkers related to the deregulation of lipid metabolism may offer advances in diagnosis of the disease. In our study three modified proteins: apolipoprotein E (Apo E), zinc-α-2-glycoprotein, and apolipoprotein A-IV, implicated in lipid homeostasis, were found. apolipoprotein A-IV (Apo A-IV), a glycoprotein formed in intestine and then released in plasma, showed an increased oxidation in patients in relapsing phase compared with both remitting phase and controls. ApoA-IV is associated with triglyceride-rich lipoproteins and high cholesterol lipoprotein (HDL), and is also found as a lipoprotein-free form [44]. It has been demonstrated that Apo A-IV has an important role in lipid metabolism, and a defensive function against atherosclerosis acting as endogenous inhibitor of lipid oxidation [45]. Moreover, considering its role in the myelination [44], [46], the oxidative damage of Apo A-IV could cause myelin dysfunction, thus contributing to demyelination process characteristic of MS. Interestingly, since Apo A-IV was found to be more oxidized in relapse compared to healthy patients, and to patients in remitting phase, it underlies that relapsing phase is characterized by high OS levels, in contrast to remitting phase that seems to be indistinguishable from controls. This result is in line with the above reported results on total protein carbonyl levels, and led us to hypothesize that Apo A-IV may be considered a putative biomarker to predict the occurrence of relapses.

By the comparison of serum proteome profile of MS patients and healthy controls, cytoskeletal proteins, such as nebulin-related anchoring protein and gelsolin, were found increasingly oxidized in relapse compared to controls. Gelsolin is a cytoplasmic actin-binding protein involved in actin assembly and filament remodeling [47]. It is also known to have an extracellular isoform called plasma gelsolin [48]. The main investigated role of gelsolin is its extracellular actin scavenging activity, resulting in actin removal from plasma with consequent consumption of gelsolin [49]. During MS, axonal degeneration leads to a release of actin within the CNS, which may mobilize gelsolin from plasma to CNS [50], as demonstrated by studies showing low levels of gelsolin in blood and in CSF of MS patients [50], [51]. In contrast, in a recent study gelsolin was found over-expressed in CSF of MS patients [31], thus suggesting that during the development of the disease there may be a change in the concentration of some proteins [50]. Furthermore, gelsolin inhibits apoptosis [52], and, during physiological conditions, binds amyloid-β reducing its amount and preventing its fibrillation [53]. In light of these considerations, we propose that oxidized gelsolin may have a patho-physiological relevance in MS, including axonal degeneration and alteration of amyloid-β metabolism.

In autoimmune diseases, such as MS, there is a substantial increment of apoptotic cells [54], and an impairment of their clearance. In our study, serum amyloid P component (SAP) was increasingly oxidized during relapsing phase. Considering the SAP role in binding apoptotic cell and favoring their elimination [55], it is reasonable to propose that alteration of this protein is a specific trait of MS.

In conclusion, our findings support the hypothesis that OS plays a central role in MS. Indeed, the oxidized proteins found in MS correlate with some aspects of the clinical course of the pathology, and may be considered specific blood-based diagnostic and prognostic markers. Interestingly remission seems to be a “healthy-like” condition where apparently almost no significant accumulation of oxidative damage occurs. Accordingly, the clinical evaluation often fails to diagnose MS when patients are in the remission phase. It is reasonable to hypothesize that OS occurs at a level insufficient for immediate toxic effect. However it is still able to initiate a cascade of reactions affecting the function of several proteins that progressively become impaired and eventually culminate in relapse outburst. At this level, severe disability and inflammatory events occur and oxidative damage seems to affect multiple pathways. Further, large studies are needed to confirm our results and to support the above proposed hypotheses.

## Supporting Information

Figure S1 **Levels of protein carbonyls of high-abundant proteins.** High-abundant proteins were removed from serum of control (CTR) and remitting (REM) and relapsing (REL) multiple sclerosis patients, and assayed for protein carbonyls by western blot analysis using a pool for each group of samples as described in Material and Methods and running them in triplicate. Representative immunoblot is shown in the upper part of the figure. The bar graph shows the densitometric evaluation and values are expressed as mean ± SD (*p = 0,02 REL vs CTR).
(TIF)

Figure S2 **2D-gels.** Three different 2D-gels for every group of analysis are shown (control, CTR; remitting, REM; and relapsing REL). Differentially expressed proteins are circled and labeled with their names.
(TIF)
