# Supplementary figures and images for: Involvement of Oxidative Stress in Occurrence of Relapses in Multiple Sclerosis: The Spectrum of Oxidatively Modified Serum Proteins Detected by Proteomics and Redox Proteomics Analysis
Source: PLoS One. 2013 Jun 7;8(6):e65184. doi: 10.1371/journal.pone.0065184 (PMC3676399; doi:10.1371/journal.pone.0065184)

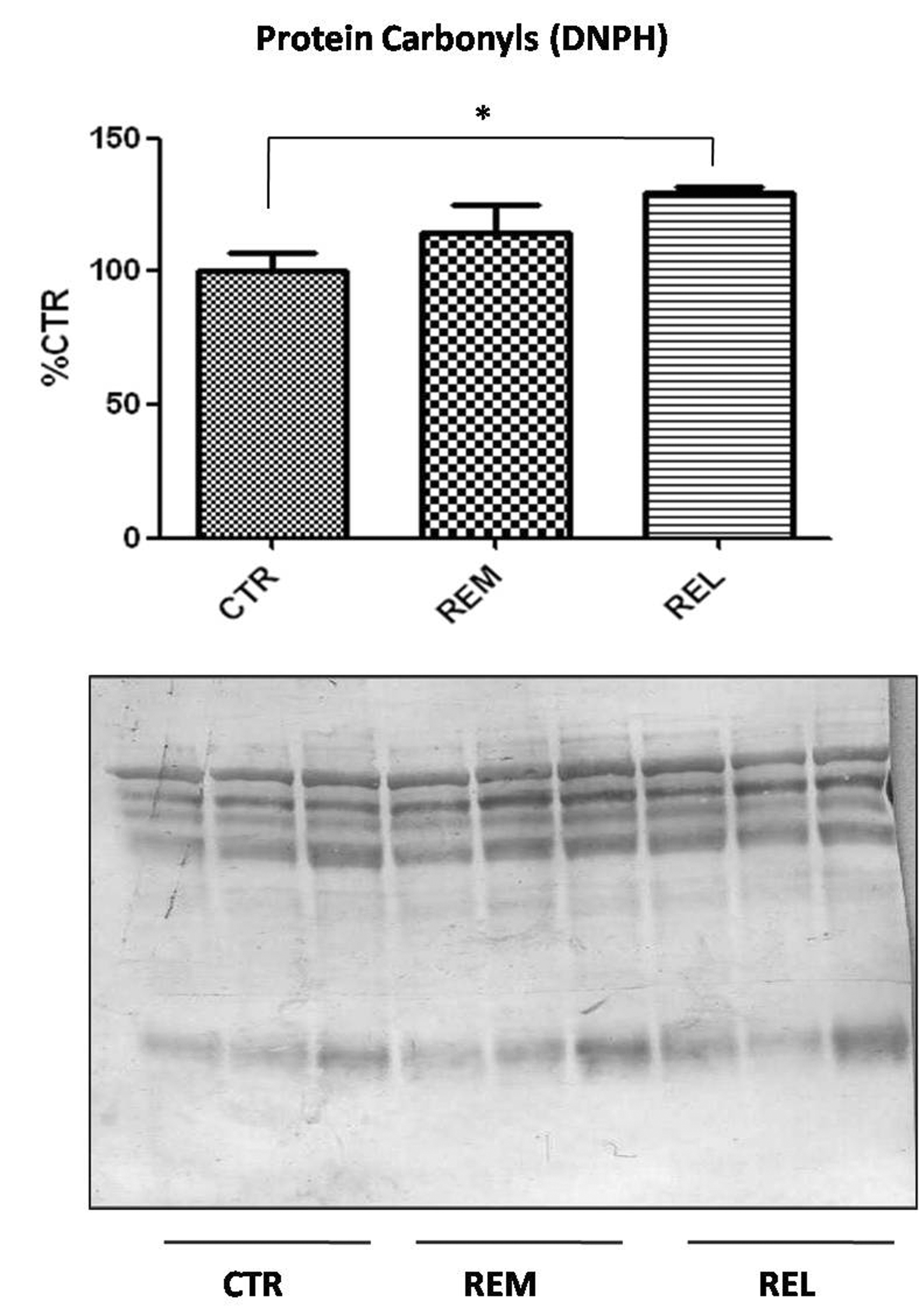

Supplement: Figure S1 — Levels of protein carbonyls of high-abundant proteins. High-abundant proteins were removed from serum of control (CTR) and remitting (REM) and relapsing (REL) multiple sclerosis patients, and assayed for protein carbonyls by western blot analysis using a pool for each group of samples as described in Material and Methods and running them in triplicate. Representative immunoblot is shown in the upper part of the figure. The bar graph shows the densitometric evaluation and values are expressed as mean ± SD (*p = 0,02 REL vs CTR). (TIF) [file pone.0065184.s001.tif]

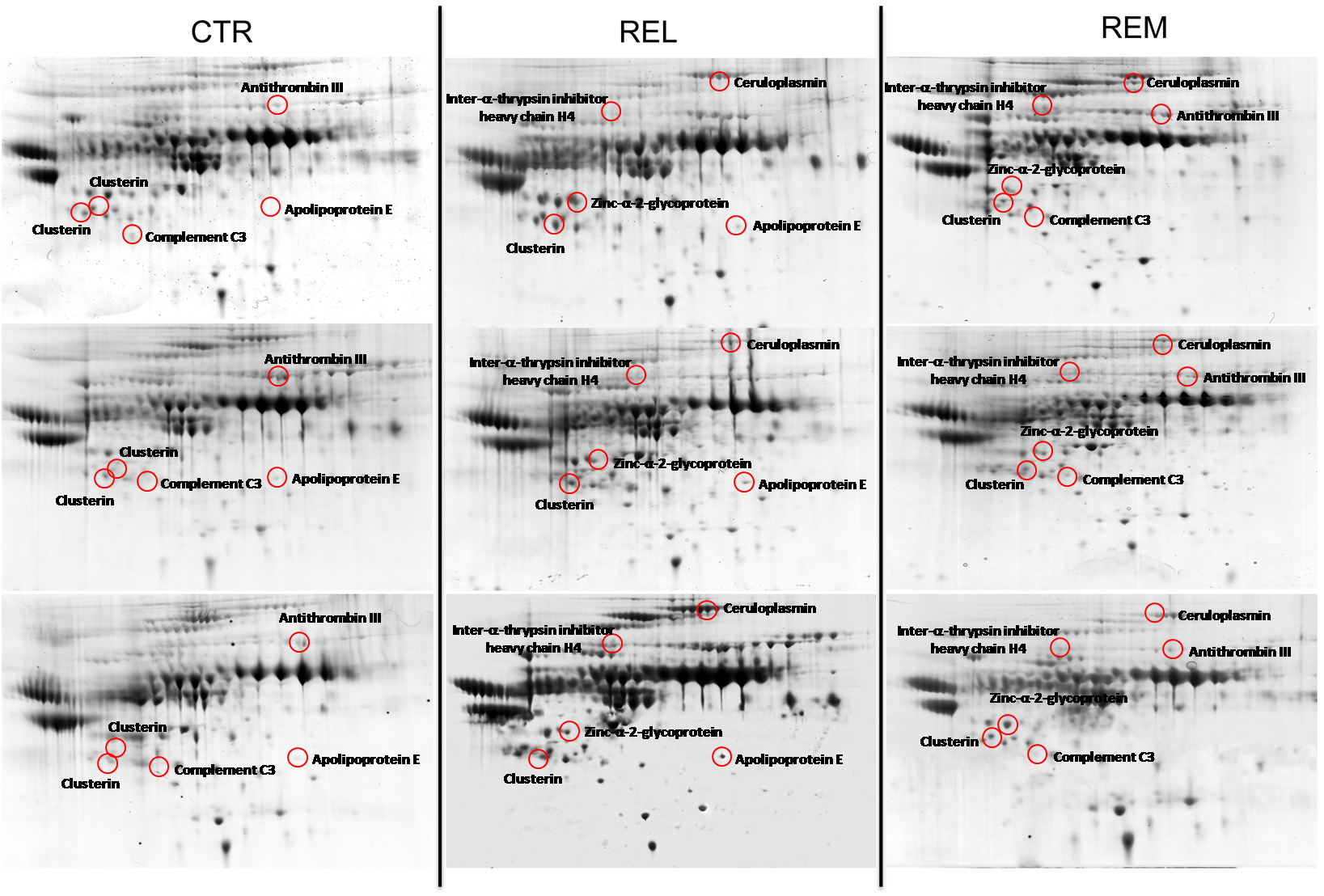

Supplement: Figure S2 — 2D-gels. Three different 2D-gels for every group of analysis are shown (control, CTR; remitting, REM; and relapsing REL). Differentially expressed proteins are circled and labeled with their names. (TIF) [file pone.0065184.s002.tif]
